# Optimal CT perfusion thresholds for core and penumbra in acute posterior circulation infarction

**DOI:** 10.3389/fneur.2023.1092505

**Published:** 2023-02-09

**Authors:** Leon Stephen Edwards, Cecilia Cappelen-Smith, Dennis Cordato, Andrew Bivard, Leonid Churilov, Longting Lin, Chushuang Chen, Carlos Garcia-Esperon, Kenneth Butcher, Tim Kleinig, Phillip M. C. Choi, Xin Cheng, Qiang Dong, Richard I. Aviv, Mark William Parsons

**Affiliations:** ^1^Department of Neurology and Neurophysiology, Liverpool Hospital, Sydney, NSW, Australia; ^2^South Western Sydney Clinical School, University of New South Wales, Sydney, NSW, Australia; ^3^Sydney Brain Centre, Ingham Institute of Applied Medical Research, Sydney, NSW, Australia; ^4^School of Medicine and Public Health, University of Newcastle, Newcastle, NSW, Australia; ^5^Melbourne Brain Centre at the Royal Melbourne Hospital, University of Melbourne, Parkville, VIC, Australia; ^6^Department of Neurology, John Hunter Hospital, Newcastle, NSW, Australia; ^7^Stroke and Brain Injury Group, Hunter Medical Research Institute and the University of Newcastle, Newcastle, NSW, Australia; ^8^Prince of Wales Clinical School, University of New South Wales, Sydney, NSW, Australia; ^9^Department of Neurology, Royal Adelaide Hospital, Adelaide, SA, Australia; ^10^Department of Neurosciences, Box Hill Hospital, Eastern Health Clinical School, Monash University, Melbourne, VIC, Australia; ^11^Department of Neurology, Huashan Hospital, Fudan University, Shanghai, China; ^12^Division of Neuroradiology, Department of Radiology, University of Ottawa and The Ottawa Hospital, Ottawa, ON, Canada

**Keywords:** ischaemic stroke, CT perfusion (CTP), computerized tomography (CT), magnetic resonance imaging, cerebral perfusion, posterior circulation infarction

## Abstract

**Background:**

At least 20% of strokes involve the posterior circulation (PC). Compared to the anterior circulation, posterior circulation infarction (POCI) are frequently misdiagnosed. CT perfusion (CTP) has advanced stroke care by improving diagnostic accuracy and expanding eligibility for acute therapies. Clinical decisions are predicated upon precise estimates of the ischaemic penumbra and infarct core. Current thresholds for defining core and penumbra are based upon studies of anterior circulation stroke. We aimed to define the optimal CTP thresholds for core and penumbra in POCI.

**Methods:**

Data were analyzed from 331-patients diagnosed with acute POCI enrolled in the International-stroke-perfusion-registry (INSPIRE). Thirty-nine patients with baseline multimodal-CT with occlusion of a large PC-artery and follow up diffusion weighted MRI at 24–48 h were included. Patients were divided into two-groups based on artery-recanalization on follow-up imaging. Patients with no or complete recanalisation were used for penumbral and infarct-core analysis, respectively. A Receiver operating curve (ROC) analysis was used for voxel-based analysis. Optimality was defined as the CTP parameter and threshold which maximized the area-under-the-curve. Linear regression was used for volume based analysis determining the CTP threshold which resulted in the smallest mean volume difference between the acute perfusion lesion and follow up MRI. Subanalysis of PC-regions was performed.

**Results:**

Mean transit time (MTT) and delay time (DT) were the best CTP parameters to characterize ischaemic penumbra (AUC = 0.73). Optimal thresholds for penumbra were a DT >1 s and MTT>145%. Delay time (DT) best estimated the infarct core (AUC = 0.74). The optimal core threshold was a DT >1.5 s. The voxel-based analyses indicated CTP was most accurate in the calcarine (Penumbra-AUC = 0.75, Core-AUC = 0.79) and cerebellar regions (Penumbra-AUC = 0.65, Core-AUC = 0.79). For the volume-based analyses, MTT >160% demonstrated best correlation and smallest mean-volume difference between the penumbral estimate and follow-up MRI (*R*^2^ = 0.71). MTT >170% resulted in the smallest mean-volume difference between the core estimate and follow-up MRI, but with poor correlation (*R*^2^ = 0.11).

**Conclusion:**

CTP has promising diagnostic utility in POCI. Accuracy of CTP varies by brain region. Optimal thresholds to define penumbra were DT >1 s and MTT >145%. The optimal threshold for core was a DT >1.5 s. However, CTP core volume estimates should be interpreted with caution.

## Introduction

Approximately 20% of ischaemic strokes involve the posterior circulation (1, 2). The posterior circulation has a unique anatomy and physiology distinguishing it from the anterior circulation (3). Compared to anterior circulation ischaemic stroke (ACS), posterior circulation infarction (POCI) is frequently misdiagnosed (4, 5), experience delayed treatment (6) and may result in worse long term functional outcome (7). POCI often presents with non-specific symptoms including headache, dizziness and gait disturbance, making the diagnosis difficult on clinical grounds alone (5). Studies have reported that up to 90% of POCI do not meet the typical clinical criteria for TIA at first medical contact (5, 8). In addition, POCI are poorly detected by clinical screening tools such as the Face Arm Speech test (FAST) score (8). Despite being underrepresented in clinical trials (8, 9), POCI benefit from acute reperfusion therapies (10, 11).

Targeted selection based on delineation of the irreversibly damaged infarct core and critically hypoperfused penumbra has underpinned many recent acute reperfusion trials. Accurate determination of the extent of these tissues and quantification of “mismatch” has been integral to expanding eligibility and extending the therapeutic time window for thrombolysis (12, 13) and mechanical thrombectomy (14, 15) in ACS. CT perfusion (CTP) facilitates rapid and accurate determination of infarct core and penumbral volumes (16). Guidelines now recommend using CTP to tailor treatment to tissue characteristics rather than strict time base criteria (17). Multiple studies have explored the optimal CTP thresholds to quantify core (18) and penumbra (19, 20) in ACS. To date, there are no studies which quantify the optimal perfusion thresholds for POCI (21). We aimed to define the optimal CTP thresholds for core and penumbra in POCI.

## Methods

### Patients

Data were analyzed from consecutive patients diagnosed with acute posterior circulation infarction enrolled in the International Stroke Perfusion Imaging Registry (INSPIRE). Patients were recruited from nine hospitals across Australia, Canada and China between February 2007 and February 2020. Patients had multimodal CT (non-contrast CT, CT angiography and CT perfusion) at acute presentation with follow up imaging (CT or MRI) performed at 24 to 48 h post stroke. Patients were treated with acute reperfusion therapies (including thrombolysis and mechanical thrombectomy) according to local eligibility protocols. Modified Rankin Scale (mRS) was documented at 90 days following stroke by an accredited assessor. Written informed consent was obtained from all participants enrolled in the registry. The INSPIRE study was approved by the local hospital ethics committees in accordance with Australian National Health and Medical Research Council guidelines.

Baseline and follow up imaging was assessed by a central imaging panel with each case being analyzed by at least two experienced readers. Cases with disagreement were resolved *via* consensus review with a third reader. Occlusion severity on baseline CT angiography (CTA) was graded using the thrombolysis in myocardial infarction flow grade score (TIMI) in keeping with previously published methods (19). Patients were included if they had evidence of complete occlusion on baseline CTA represented by a TIMI of 0 in a branch of the verterobasilar circulation. Patients were excluded due to the following reasons including; no follow up 24–48 h diffusion weighted MRI, missing CTP data, inadequate CTP scan coverage of the posterior circulation, excessive movement during CTP, absence of complete occlusion (TIMI 0) of a posterior circulation artery (defined as vertebral, basilar or posterior cerebral arteries) on baseline CT angiography, or partial recanalization (TIMI 1–2) on follow up vessel imaging.

Patients were divided into two groups on the basis of occlusion severity on follow up CTA or magnetic resonance angiography (MRA) performed at 24 to 48 h post stroke as adjudicated by the central imaging panel. Group 1 demonstrated evidence of complete occlusion of a posterior circulation artery on follow up imaging, defined by a TIMI of 0. This group was used to represent the acute penumbral lesion. Group 2 demonstrated “major reperfusion” with complete revascularization of the posterior circulation, defined by a TIMI score of 3 on follow up imaging, and was used to represent the acute infarct core. The selection process is summarized in Supplementary Figure 1.

### Image acquisition

Baseline CT imaging was performed using 64-, 128-, 256-, or 320- detector scanners. Axial z-axis coverage ranged from 40 to 160 mm. Scan acquisition sequence was; a non-contrast helical CT from skull base to vertex followed by CTP and then CTA from aortic arch to vertex. Details of each scanner and CTP protocol are provided in Supplementary Table 1.

Follow up MRI was performed at 24 to 48 h post stroke irrespective of treatment using 1.5 to 3 tesla scanners. The MRI protocol included an axial gradient-echo T2^*^-weighted series, isotropic diffusion-weighted imaging (DWI), MR time of flight angiography and fluid-attenuated inversion recovery imaging.

### CTP post-processing

CTP maps were calculated using a commercial image processing package (AutoMiStar, Apollo medical imaging technologies, Melbourne, Australia). This software automatically performs motion correction. It then autonomously derives arterial input and venous output functions by selecting an unaffected major artery (commonly the anterior cerebral artery) and venous sinus (commonly the superior sagittal sinus). Selected inputs were confirmed by an expert analyst (LE) prior to image processing. Areas of gliosis, chronic infarction and cerebrospinal fluid were automatically masked from perfusion maps using a Hounsfield unit threshold. CTP source imaging was processed using delay and dispersion corrected singular value decomposition deconvolution (24). Processed maps included delay time to peak of the residue function (DT), mean transit time (MTT), cerebral blood flow (CBF) and cerebral blood volume (CBV). This method of deconvolution produces delay time maps rather than the standard time to peak of the residue function maps (Tmax).

### Imaging analysis

Each CTP source imaging slab was coregistered a 3d rigid method to the corresponding 24 h DWI using the MiStar imaging fusion tool. Accurate co-registration was confirmed using interactive visual blending (L.E.). Regions of interest representing the posterior circulation vascular territory and specific vascular territories of the thalamoperforating/basilar tip, basilar perforating, calcarine, non-calcarine posterior cerebral and cerebellar arteries were determined using a template (22) (Supplementary Figure 1) which was manually adjusted to the CTP source imaging. The 24 h DWI lesion was semi-automatically delineated using a region of interest tool based on signal intensity thresholds. Regions of interest were transferred to the acute CTP maps. A range of relative CTP thresholds were incrementally investigated (Supplementary Table 2). Relative thresholds were defined respective to the mean perfusion values for tissue in the contralateral hemisphere.

### Statistical analysis

Data processing, analyses and visualization were performed using Python (version 3.9) using the NumPy, SciPy, and Matplotlib packages. Receiver operating curve (ROC) analysis was used to investigate the optimal CTP parameter threshold relative to follow up DWI infarct volume. Optimality was defined as the threshold and parameter which maximized the area under the curve (AUC). The DWI lesion was considered the “true” lesion. Overlapping voxels between the DWI lesion and the CTP lesion were considered “true positive.” DWI voxels outside of the overlapping DWI and CTP lesions were considered “true negative.” Voxels within the CTP lesion but not in the DWI lesion were considered “false positive.” Voxels within the DWI lesion but not within the CTP lesion were labeled as “false negative.” Sensitivity [true positive/(true positive + false negative)] was plotted against 1 - specificity [true negative/(true negative + false positive)] to generate a ROC curve for each perfusion map. An area under the curve (AUC) was determined for each ROC curve. Sensitivity, specificity, positive predictive value and negative predictive value were calculated for each perfusion threshold increment. Area under the curve was calculated for each using the ROC. An AUC of < 0.7 was considered poor for this study.

Only voxels within the posterior circulation vascular territory template were included in the analyses. This prevents a large number of “true negative” voxels relative to “false negative” voxels from disproportionately influencing the calculation of specificity and AUC calculation. Volume analysis was performed by scrutinizing the threshold which produced the smallest mean volume difference between the acute perfusion lesion and follow up imaging. Linear regression was used to assess the relationship between the acute CTP lesion and follow up MRI volumes. Agreement with the following DWI lesion was assessed (Bland-Altman) at the perfusion threshold resulting in the smallest mean volume difference.

### Data availability

Individual patient data from INSPIRE is not publically available. Individual data can be shared with partners based upon individual transfer agreements upon request with the corresponding author.

## Results

### Patients

There were 331 patients with POCI in the INSPIRE across the assessed period. Exclusions are detailed in the STAndards for the Reporting of Diagnostic accuracy studies (STARD) diagram (Supplementary Figure 1). Thirty nine patients were included for analysis in this study. Median age was 71 years (range 34–91 years) and 41% were female. Median National Institute of Health Stroke Scale (NIHSS) score was 11 (interquartile range [IQR]: 6–17).

Median last known well (LKW) time to CTP was 223 (IQR: 109–385) min. Median time to follow up imaging was 1.8 days (IQR: 1.1–3.7 days). On follow up imaging, 12 patients (30.8%) had no reperfusion (Group 1) and 27 patients (69.2%) had major reperfusion (Group 2). Thrombolysis was administered in 23 patients (59.0%). Thrombectomy was performed in 21 patients (53.8%). Eleven patients (28.2%) received both therapies. Compared to patients with no reperfusion (Group 1), those with major reperfusion (Group 2) had higher rates of acute treatment including thrombolysis (18/27 [66.7%] vs. 5/12 [41.7%]), thrombectomy (17/27 [63.0%] vs. 4/12 [33.3%]) or combined therapy (9/27 [33.3%] vs. 2/12 [16.7%]). This difference was not significant (all *p* > 0.05). Median LKW time to thrombolysis and thrombectomy was 189 (IQR: 137–295) and 415 (IQR: 249–547) min, respectively.

### Group 1: Penumbral analysis of patients with persistent complete occlusion of a posterior circulation artery on follow up vessel imaging

**Figure 1:**
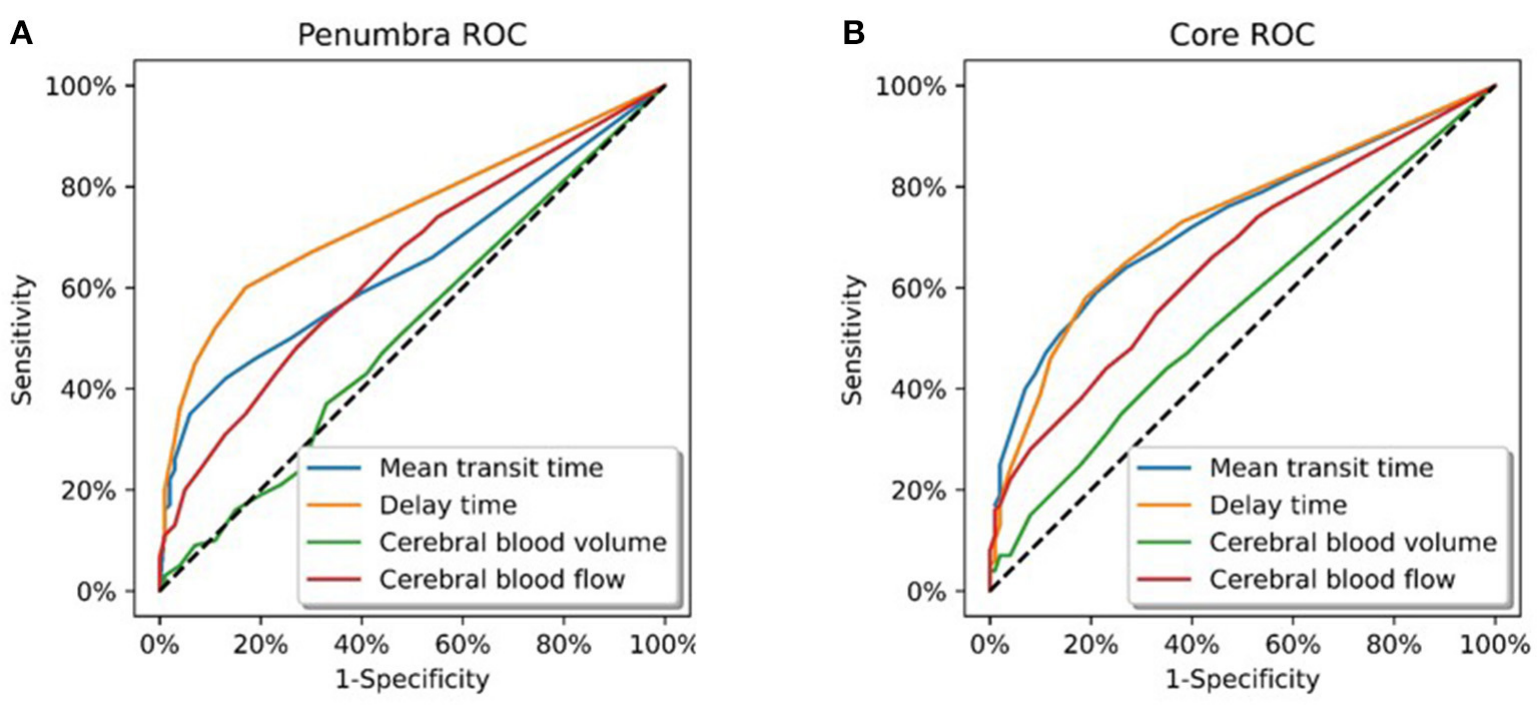
Receiver operating curve analysis. **(A)** All patients in Group 1 (total of 6258358 voxels). These patients demonstrated a complete occlusion of a posterior circulation vessel on follow up imaging. Delay time was the CT parameter which optimally represented the ischaemic penumbra (AUC = 0.74). **(B)** All patients in Group 2 (total of 12436400 voxels). These patients demonstrated major reperfusion (TIMI 3) of the initially occluded posterior circulation vessel on follow up imaging. Delay time and mean transit time were the CT parameters which optimally represented the infarct core (AUC = 0.73).

Of the 12 patients in this group; there were two vertebral, two basilar, and eight posterior cerebral artery occlusions. Voxel based ROC analysis of all patients in the group (Figure 1A) demonstrated the perfusion parameter with the maximal AUC was delay time (AUC 0.74). The optimal delay time threshold for predicting penumbra was a DT >1 s (sensitivity 0.60, specificity 0.83) (Table 1). The optimal thresholds for the other perfusion parameters were: MTT >145% (sensitivity 0.35, specificity 0.94), CBF < 75% (sensitivity 0.53, specificity 0.68) and CBV < 80% (sensitivity 0.37, specificity 0.67).

**Table 1:** Optimal perfusion thresholds to define penumbra and core according to voxel analysis.

**Optimal Threshold map**	**Area under curve**	**Sensitivity**	**Specificity**	**Positive predictive value**	**Negative predictive value**
**Group 1—Penumbra**					
Delay time >1 s	0.71	0.60	0.83	0.19	0.97
Mean Transit time >145%	0.65	0.35	0.94	0.28	0.96
Relative cerebral blood flow < 75%	0.61	0.53	0.68	0.10	0.96
Relative cerebral blood volume < 80%	0.52	0.10	0.68	0.07	0.94
**Group 2—Core**					
Delay time >1.5 s	0.70	0.58	0.81	0.17	0.97
Mean transit time >145%	0.68	0.51	0.86	0.19	0.96
Relative cerebral blood flow < 45%	0.60	0.28	0.92	0.19	0.95
Relative cerebral blood volume < 70%	0.54	0.31	0.7	0.08	0.95

**Figure 2:**
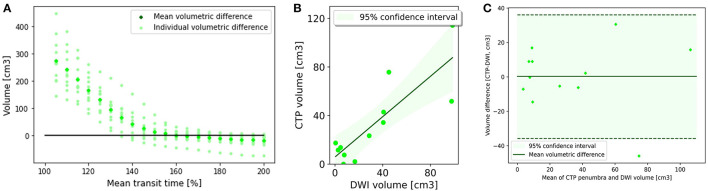
Penumbral analysis. **(A)** Mean volumetric difference between follow up diffusion weighted imaging lesion and acute perfusion lesion according to mean transit time threshold. In Group 1, the perfusion lesion identified at a MTT of >160% resulted in the smallest volumetric difference with follow up MRI (0.31 ml3, CI −11.98 to 12.53). **(B)** CTP lesion volume using Mean transit time >160% vs. 24 h DWI lesion volume. There was good correlation between the acute perfusion lesion and follow up imaging volume (*R*2 = 0.71). **(C)** Bland-Altman plot demonstrating the volumetric agreement between the perfusion lesion at Mean Transit time >160% and follow up DWI. There is reasonable agreement with the majority of cases (11 of 12) within the 95% confidence interval.

Volumetric analysis indicated MTT >160% was best correlated with 24 h infarct volume, with an *R*^2^ of 0.71 (Table 2; Figures 2A, B). This led to a mean volumetric difference (MVD) between the acute perfusion and 24 h MRI lesion of 0.31 cm^3^ (95% CI −11.92 to 12.53). The Bland-Altman 95% limits of agreement were-37.75 and 36.04 cm^3^ (Figure 2C). This was followed by DT >2.5 s (*R*^2^ = 0.41), CBV < 50% (*R*^2^ = 0.17), and CBF < 40% (*R*^2^ = 0.01).

**Table 2:** Optimal perfusion thresholds to define penumbra and core according to volume analysis.

**Optimal threshold map**	**Mean difference, cm^3^ (95% Cl)**	***R* ^2^**
**Group 1—Penumbra**		
Mean transit time >160%	0.31 (−11.92 to 12.53)	0.71
Delay time >2.5 s	0.32 (−16.53 to 17.17)	0.41
Relative cerebral blood volume < 40%	2.11 (−24.42 to 20.21)	0.002
Relative cerebral blood volume < 50%	4.27 (−24.31 to 32.84)	0.17
**Group 2—Core**		
Mean transit time >170%	0.03 (−13.19 to 13.24)	0.11
Relative cerebral blood volume < 45%	−2.01 (−23.36 to 8.98)	< 0.01
Delay time >4.5 s	−2.60 (−16.75 to 11.56)	0.03
Relative cerebral blood flow < 40%	−3.93 (16.35 to 8.45)	0.13

cm^3^, cubic centimeters; 95% CI, Confidence Interval.

Analysis of posterior circulation subregions demonstrated that CTP was most accurate in the calcarine territory (AUC = 0.77) (Table 3) with the smallest MVD (0.24 cm^3^, 95% CI −18.91 to 19.38) (Supplementary Table 3). CTP penumbral estimates were less accurate in the other POCI subregions including the cerebellar (AUC = 0.66, MVD 0.95 cm^3^), non-calcarine PCA (AUC = 0.61, MVD 1.08 cm^3^), thalamoperforator and basilar tip (AUC = 0.55, MVD −1.42 cm^3^) and basilar perforating artery (AUC = 0.53, MVD 1.48 cm^3^). CTP tended to overestimate the acute penumbral volume within regions other than the thalamoperforator and basilar tip vascular regions.

**Table 3:** Optimal perfusion thresholds to define penumbra and core according to voxel analysis by subregion.

Subregion^*^	**Optimal threshold map**	**Number of patients**	**Area under curve**	**Sensitivity**	**Specificity**	**Positive predictive value**	**Negative predictive value**
**Group 1—Penumbra**					
Calcarine	DT >1 secs	7	0.75	0.69	0.80	0.15	0.97
Cerebellar	DT >0.5 secs	5	0.65	0.65	0.70	0.03	0.99
Non-calcarine PCA	MTT >135%	7	0.61	0.45	0.78	0.02	0.99
Basilar perforating	CBF < 95%	2	0.53	0.57	0.50	< 0.01	1
Thalamo-perforating and basilar tip	MTT >145%	9	0.55	0.22	0.88	0.03	0.99
**Group 2—Core**					
Calcarine	MTT >135%	14	0.79	0.81	0.77	0.14	0.99
Cerebellar	DT >1.5 secs	15	0.79	0.81	0.77	0.09	0.99
Non-calcarine PCA	MTT >150	11	0.64	0.44	0.84	0.04	0.99
Basilar perforating	DT >1.5 secs	10	0.73	0.74	0.72	< 0.01	1
Thalamo-perforating and basilar tip	DT >9.5 secs	12	0.50	< 0.01	1	< 0.01	1

^*^Subregion refers to the area supplied by the corresponding artery. MTT, Mean transit time, DT, Delay time; CBV, Relative cerebral blood volume; CBF, Relative cerebral blood flow; Secs, seconds.

### Group 2: Core analysis of patients with complete recanalization of an initially occluded posterior circulation artery on follow up vessel imaging

Of the 27 patients in this group; there were 4 vertebral, 17 basilar, and 6 posterior cerebral artery occlusions. On voxel based analysis of all patients in the group (Figure 1B), both DT and MTT demonstrated the highest AUC (0.73). The optimal threshold for these parameters were a MTT >145% (sensitivity 0.51, specificity 0.86) and DT >1.5 s (sensitivity 0.58, specificity 0.81). The optimal perfusion thresholds for the other parameters were: CBF < 45% (sensitivity 0.28, specificity 0.92) and CBV 70% (sensitivity 0.31, specificity 0.77).

**Figure 3:**
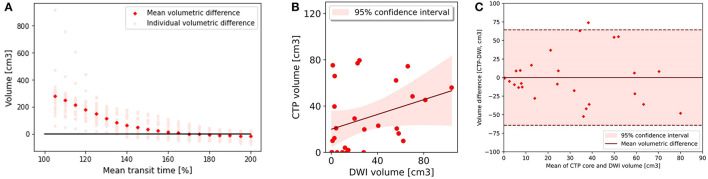
Core analysis. **(A)** Mean volumetric difference between follow up diffusion weighted imaging lesion and acute perfusion lesion according to mean transit time threshold. In group 2, the perfusion lesion identified at a MTT of >170% resulted in the smallest volumetric difference with follow up MRI 0.03 (−13.19 to 13.24). **(B)** CTP lesion volume using Mean transit time >170% versus 24 h DWI lesion volume. There was poor correlation between the acute perfusion lesion and follow up imaging volume (*R*^2^ = 0.11). **(C)** Bland-Altman plot demonstrating the volumetric agreement between the perfusion lesion at Mean Transit time >170% and follow up DWI. There is reasonable agreement with the majority of cases (26 of 27) within the 95% confidence interval.

A MTT of > 170% led to the smallest MVD with the follow up infarct volume (0.03 cm^3^, 95% CI −13.19 to 13.24) (Figures 3A, B) (Table 2). The Bland-Altman 95% limits of agreement were-64.24 and 64.30 cm^3^ (Figure 3C). All acute perfusion parameters demonstrated low correlation with follow up infarct volume (MTT >170% *R*^2^ 0.11, CBF < 40% *R*^2^ 0.13, DT >4.5 s *R*^2^ 0.03, and CBV < 45% *R*^2^ 0.01).

On subregion analysis, CTP was most accurate in detection of calcarine and cerebellar lesions with an AUC of 0.79 in both areas (Table 3). This was followed by basilar perforating (AUC 0.73), non-calcarine PCA (0.64) and thalamoperforators and basilar tip regions (0.50). DT and MTT were the optimal CTP parameters in these regions.

## Discussion

Our results demonstrate the diagnostic utility of CTP in patients with POCI. CTP accuracy varies by brain region with better performance in the calcarine and cerebellar areas. Our results suggest that commonly used perfusion thresholds are suboptimal for characterization of the penumbra and core in the posterior circulation (Supplementary Table 4). As such, thresholds used in commercial CTP software packages should be interpreted with caution when making clinical decisions in suspected POCI.

The optimal perfusion thresholds for POCI detection were: DT for penumbra (AUC = 0.74) and both MTT and DT for core (AUC = 0.73). Voxel analysis of individual thresholds demonstrated that a DT of >1 and >1.5 s best represented core (AUC = 0.71) and penumbra (AUC = 0.70), respectively. Threshold accuracy was lower than that previously reported for anterior circulation strokes (AUC 0.86–0.93) (20, 23, 24). However, accuracy was comparable with previous studies which have examined the combined utility of Non-contrast CT, CT angiography source imaging and raw CTP perfusion map analysis to detect POCI (AUC 0.64–0.86) (25, 26).

Compared to cortical regions, subcortical strokes are not readily identified by CTP (27, 28). Despite this, CTP improves stroke diagnosis in subcortical regions and posterior circulation compared to inspection of NCCT alone (25, 27, 28). Furthermore, studies have shown that CTP better predicts functional outcome than clinical parameters or imaging scores based on CTA source imaging or NCCT (29). We found that core and penumbra in the basilar perforating, basilar tip and thalamoperforating subregions, in particular, were poorly estimated using CTP. Within these areas there was variability in the optimal perfusion parameter and threshold. This is likely in part related to the small sample of patients with strokes in these areas. As such there was not a single “optimal” perfusion parameter or threshold encompassing all POCI subregions. Additional studies of these specific subregions are warranted, including different deconvolution methods and machine learning approaches, which may improve estimates in these areas.

Multiple studies have demonstrated that expert assessment of raw CTP maps in suspected POCI improves the sensitivity and diagnostic accuracy over examination of automated core-penumbra maps alone (26–28). A study by Capasso et al. (30) demonstrated a 93.2% false negative detection rate for POCI when using automated software based on the traditional core threshold of a relative CBF < 30%. This false negative rate was higher than inspection of non-contrast CT alone (70.4%). The false negative rate dropped to 9.1% with expert assessment of raw Tmax maps. These results imply subthreshold perfusion deficits not identified on automated core penumbra maps based on cut-off values established in the anterior circulation. Authors of these studies have advocated for expert inspection of raw perfusion maps to avoid missing treatable lesions. However, timely access to specialist neurology and radiology services is frequently constrained, particularly in regional and rural settings (28, 31). Refining the thresholds for automated core-penumbra estimates may allow more patients in limited resource settings to receive timely diagnosis and treatment.

The optimal thresholds derived in our study are “shorter” than the set points used for automated core-penumbra maps outputted by CTP software including the imaging tool used in this analysis. Previous studies (19, 20) have shown cerebral blood flow < 30% and a delay time >3 s as accurate representations of core and penumbra, respectively. Importantly, the current thresholds have been derived from ACS patients. Shorter thresholds have the effect of improving lesion detection sensitivity but reducing specificity. CTP has higher specificity for infratentorial stroke compared to supratentorial strokes (32). As such sacrificing a degree of specificity may be valid in suspected POCI. Despite a moderate AUC, we propose that these “shorter” thresholds hold significant clinical utility, identifying additional subthreshold strokes not detected by current core-penumbra maps.

Posterior circulation vascular supply is complex. There is significant variability in the volume and spatial distribution of the brain supplied by the posterior circulation. Bonkoff et al. (22) describes 21 distinct ischaemic patterns based upon vascular supply. This detailed approach to stroke location description has been shown to better predict functional outcome. This model formed the basis of our subanalysis of distinct anatomical regions within the posterior circulation. Studies describe the variable topographic risk of infarction by brain region following posterior circulation occlusion (22, 33). These studies have demonstrated that the medial occipital region supplied by the calcarine artery carries the highest risk of future infarction (33). Our study found that of the different brain regions, CTP was most accurate in identifying penumbra and core within the calcarine and cerebellar vascular territories. These data support previous studies which have found CTP accuracy for POCI improves with larger infarcts and cerebellar location (26).

CTP penumbral volume estimates demonstrated good correlation with follow up imaging (*R* (2) 0.71). In contrast, core estimates were poorly correlated with final infarct volume (*R*^2^ 0.11). The correlation was strongest for both core and penumbra in the calcarine and cerebellar regions. Core analysis is challenging in POCI as estimates are based upon patients who achieved vessel recanalization (Group 2). Time to reperfusion was unknown in the majority of patients (66% had not undergone thrombectomy). Recanalisation may have occurred anytime between the acute to follow up imaging (median time to follow up imaging 1.8 days). Duration of ischaemia is directly related to final infarct volume. This challenge is not easily overcome, as up to 31% of patients with POCI have a false negative result on diffusion weighted MRI within the first 24 h (34). As such, caution is advised when applying CTP core estimates to clinical decision making in excluding patients from reperfusion therapy.

There are several limitations to our study. We utilized strict inclusion criteria for defining core and penumbra consistent with previous studies (19, 20, 23, 34). Our and previously published methods have relied on vessel occlusion status on follow up imaging to define the penumbra and core analysis groups. Infarct core estimates are likely to be overestimated as the exact time of vessel recanalization demonstrated on follow up imaging, in non-thrombectomy cases, is unknown. Additionally, as posterior circulation stroke represents 20% of acute ischaemic stroke, only a subset of patients were eligible for analysis. While these criteria are necessary for robust core and penumbra estimates in POCI, they resulted in a restricted analysis cohort. Despite these restrictions, our cohort of patients is commensurate with previous studies of anterior circulation stroke (20). CTP was acquired on a variety of scanners including older 64- and 128- slice devices. These machines produce a narrower z-axis coverage than 320-slice scanners. Compared to whole brain CTP, limited scan range results in greater inaccuracy in core and penumbra estimates (19). Additionally, co-registration of limited range CTP data to MRI introduces the potential for inaccuracy in stroke lesion segmentation. We used one commercial imaging package to process CTP data. Commercial packages utilize specific deconvolution algorithms and CTP parameter thresholds which are not directly comparable between proprietors. Lesion volume varies considerably by software package (19, 24). Separate packages will require specific investigation to determine optimal POCI perfusion thresholds. There were few patients in many of the subregions, this affects the robustness of our estimates in these areas. Further analysis with a larger patient cohort is warranted. POCI can take up to 72 h to become apparent on DWI (35). Median time to follow up imaging was 1.8 days in our study. As such, our DWI lesion may underrepresent the true final infarct volume in POCI. Our results are derived from a single dataset; validation of these thresholds in an external dataset is warranted.

## Conclusion

CTP is diagnostically useful in POCI. Current anterior circulation thresholds are suboptimal for estimation of the acute core and penumbra regions in POCI. A delay time of >1 and >1.5 s optimally demonstrated core and penumbra respectively. Inspection of summary maps using these thresholds may aid clinical practice by identifying subthreshold lesions not identified on automated core-penumbra maps using current values. Estimates of core volume in the posterior circulation should be interpreted with caution and not be used in isolation to exclude patients from reperfusion treatments.

## Data availability statement

The raw data supporting the conclusions of this article will be made available based upon individual transfer agreements upon request with the corresponding author.

## Ethics statement

The studies involving human participants were reviewed and approved by the Hunter New England Human Research Ethics Committee. The patients/participants provided their written informed consent to participate in this study.

## Author contributions

LE was the principal author of the manuscript. CC-S, DC, AB, LC, LL, CC, CG-E, KB, TK, PC, XC, QD, and RA were involved in the drafting and revision of the manuscript. MP was involved in the conception, drafting, and revision of the manuscript. All authors contributed to the article and approved the submitted version.
