# Supplementary material for: Optimal CT perfusion thresholds for core and penumbra in acute posterior circulation infarction
Source: Front Neurol. 2023 Feb 9;14:1092505. doi: 10.3389/fneur.2023.1092505 (PMC9947562; doi:10.3389/fneur.2023.1092505)
Supplement: Supplementary file 1 [file Data_Sheet_1.docx]

**Supplement**

**Supplementary table 1 - Acute CT perfusion acquisition protocols for the nine INSPIRE sites included in this study**

| **Site** | **CT Scanner** | **Acquisitions** | **Coverage** | **Contrast** |
| --- | --- | --- | --- | --- |
| Gosford hospital, Australia | GE lightspeed VCT (64 detectors) | 2 slabs; 19 acquisitions in 54 seconds for each slab | 80 mm | 45 ml of contrast (Ultravist 370) injected at 6 ml/s for each aquisition |
| Royal Adelaide Hospital, Australia | SIEMENS SOMATOM Definition AS+ (128 detectors | 29 acquisitions in 58 seconds | 96 mm | 40 ml of contrast (Ultravist 370) injected at 6 ml/s |
| The Queen Elizabeth Hospital, Australia | Toshiba Aquilion one (320 detectors) | 19 acquisitions in 60 seconds | 160 mm | 40 ml of contrast (Ultravist 370) injected at 6 ml/s |
| Box Hill Hospital, Australia | Toshiba Aquilion one (320 detectors) | 19 acquisitions in 60 seconds | 160 mm | 40 ml of contrast (Ultravist 370) injected at 6 ml/s |
| John Hunter Hospital, Australia | Toshiba Aquilion one (320 detectors) | 19 acquisitions in 60 seconds | 160 mm | 40 ml of contrast (Ultravist 370) injected at 6 ml/s |
| The Second Affiliated Hospital of Zhejiang University, China | SIEMENS SOMATOM Definition Flash (256 detectors) | 25 acquisitions in 62 second | 100 mm | 15 ml of contrast agent (Ultravist 370) injected at 4ml/s |
| Huashan Hospital, China | Philips Brilliance iCT (256 detectors) | 12 acquisitions in 54 seconds; shuttle mode | 125 mm | 40 ml of contrast agent (Ultravist 370) injected at 5 ml/s |
| Baotou Central Hospital, China | SIEMENS SOMATOM Definition Flash (256 detectors) | 17 acquisitions in 72 second | 150 mm | 40 ml of contrast agent (Ultravist 350) injected at 5ml/s |
| Sunnybrook Medical Centre, Canada | GE lightspeed VCT (64 detectors) | 51 acquisitions in 135 seconds | 40 mm | 0.7 ml/kg (maximum to 90 ml) of iodinated contrast agent (Omnipaque, 300 mg iodine/ml) injected at 2-4 ml/seconds |

ml: millitres, ml/s: millitres per second

**Supplementary Table 2 - Range and increments of perfusion CT parameters used for receiver operating curve and volume analysis.**

| **Perfusion CT parameter** | **Range** | **Increments** |
| --- | --- | --- |
| **Delay time (seconds)** | 0-10 | 0.5 |
| **Mean transit time (%)** | 100-200 | 5 |
| **Relative cerebral blood flow (%)** | 0-100 | 5 |
| **Relative cerebral blood volume (%)** | 0-100 | 5 |

**Supplementary Table 3 - Optimal perfusion thresholds to define penumbra and core according to volume analysis by subregion.**

| **Subregion*** | **Optimal threshold map** | **Number of patients** | **Mean difference, cm^3^ (95% CI)** | **R^2^** |
| --- | --- | --- | --- | --- |
| **Group 1- Penumbra** |  | | | |
| **Calcarine** | MTT > 180% | 7 | 0.24 (-18.91 to 19.38) | 0.45 |
| **Cerebellar** | DT > 4.5 secs | 5 | 0.95 (-9.15 to 11.04) | 0.56 |
| **Non- Calcarine PCA** | DT > 5.5 secs | 7 | 1.08 (-8.10 to 10.27) | 0.09 |
| **Basilar perforating** | DT > 2.5 secs | 2 | 1.48 (-24.11 to 27.07) | <0.01 |
| **Thalamo- perforating and basilar tip** | DT > 5 secs | 9 | -1.42 (-14.92 to 12.08) | 0.06 |
| **Group 2 - Core** |  | | | |
| **Calcarine** | MTT > 180% | 14 | 0.75 (-12.81 to 14.30) | 0.18 |
| **Cerebellar** | DT > 6.5 secs | 15 | -0.14 (-9.95 to 9.67) | 0.15 |
| **Non- Calcarine PCA** | DT > 7 secs | 11 | 0.74 (-12.53 to 11.06) | 0.11 |
| **Basilar perforating** | CBF < 10% | 10 | 0.74 (-12.53 to 11.06) | 0.11 |
| **Thalamo- perforating and basilar tip** | MTT > 200 | 12 | 0.41 (-22.33 to 23.16) | 0.13 |

*Subregion refers to the area supplied by the corresponding artery. MTT: Mean transit time, DT: Delay time, CBV: Relative cerebral blood volume, CBF: Relative cerebral blood flow, Secs: seconds, cm^3^: cubic centimetres, 95% CI: Confidence Interval

**Supplementary Table 4 – Performance of commonly applied thresholds for core and penumbra derived from anterior circulation stroke.**

| **Optimal Threshold map** | **Area under curve** | **Sensitivity** | **Specificity** | **Positive predictive value** | **Negative predictive value** |
| --- | --- | --- | --- | --- | --- |
| **Group 1 - Penumbra** |  | | | | |
| **Delay time > 3 second** | 0.64 | 0.30 | 0.97 | 0.40 | 0.95 |
| **Group 2 - Core** |  | | | | |
| **Cerebral blood flow < 30%** | 0.57 | 0.16 | 0.99 | 0.42 | 0.95 |

**Supplementary Figure 1 –STandards for the reporting of Diagnostic accuracy studies (STARD) flow diagram of patient selection process.**

**
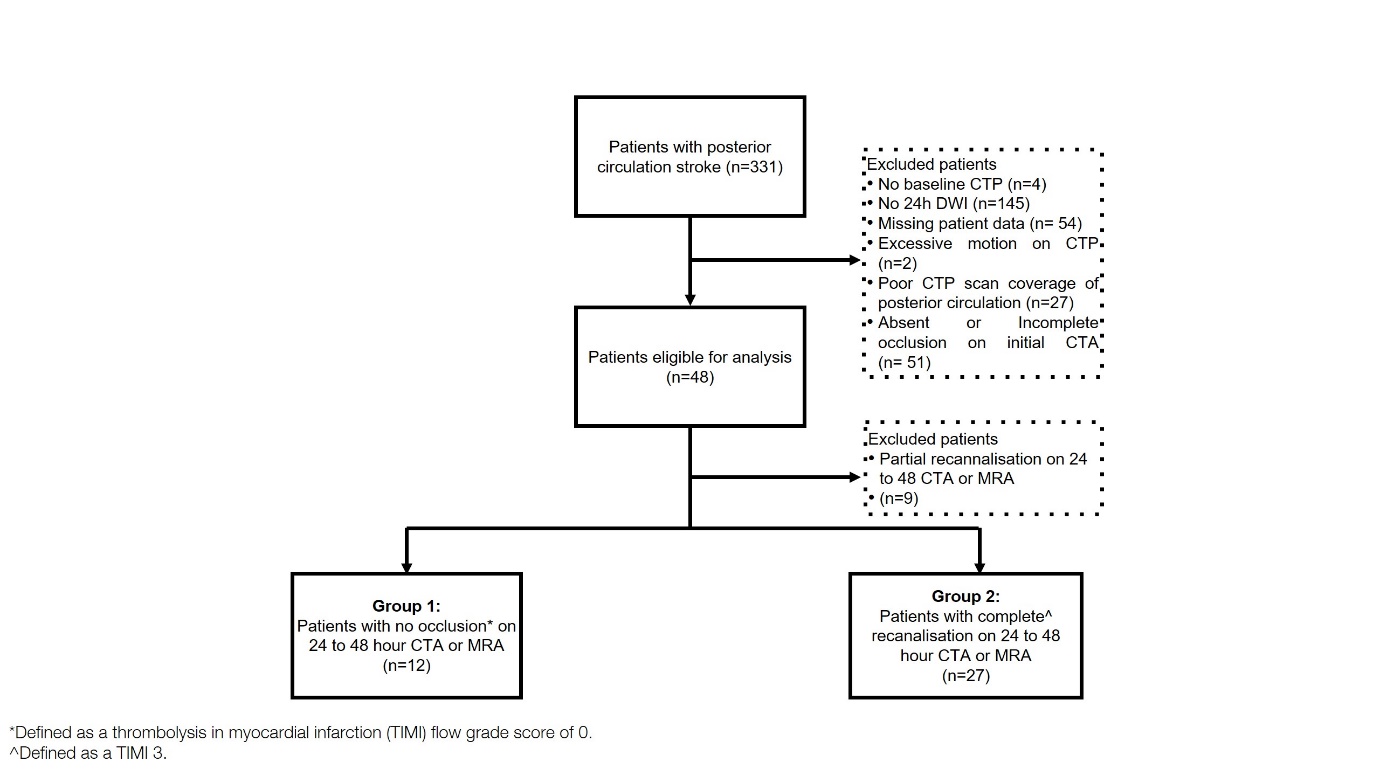
**

MRA: Magnetic resonance angiography, CTA: CT angiography, CTP: CT perfusion, DWI: Diffusion weighted imaging, 24h: 24 hours.

**Supplementary Figure 2 - Segmentation templates of PC subregions by vascular territories**

**
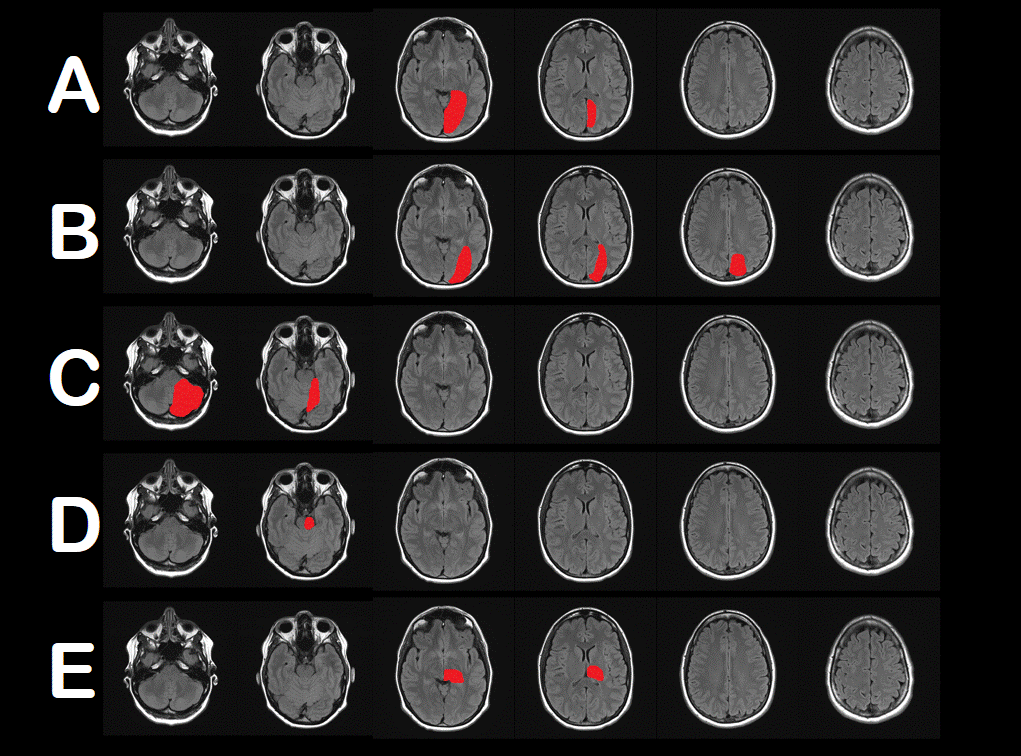
**

Illustrated areas are: (A) calcarine, (B) non-calcarine posterior cerebral artery, (C) Cerebellar, (D) basilar perforating and (E) basilar tip and thalamo-perforating artery regions. Red areas represent the segmented subregion.

**Appendix**

**Appendix: INSPIRE Study Group Co-Investigators**

| **Name** | **Location** | **Role** | **Contribution** |
| --- | --- | --- | --- |
| Ferdinand Miteff, MD | John Hunter Hospital, Newcastle, Australia | Site Investigator | Data collection |
| Congguo Yin, MD | Hangzhou First Hospital, Zhejiang University School of Medicine, Hangzhou, China | Site Investigator | Data collection |
| Peng Wang, MD | Zhejiang Provincial People’s Hospital, Hangzhou, China | Site Investigator | Data collection |
| Yu Geng, MD | Zhejiang Provincial People’s Hospital, Hangzhou, China | Site Investigator | Data collection |
| Xu Zhang, MD | The First Affiliated Hospital of Wenzhou Medical University, Wenzhou, | Site Investigator | Data collection |
| Xuezhi Yang, MD | The First Affiliated Hospital of Wenzhou Medical University, Wenzhou, China | Site Investigator | Data collection |
| Weiwen Qiu, MD | Lishui People’s Hospital, Lishui, China | Site Investigator | Data collection |
| Qi Fang, MD | The First Affiliated Hospital of Soochow University, Soochow, China | Site Investigator | Data collection |
| Yi Sui, PhD, MD | The First People’s Hospital of Shenyang, Shenyang, China | Site Investigator | Data collection |
| Wenhuo Chen, MD | Zhangzhou Municipal Hospital, Zhangzhou, China | Site Investigator | Data collection |
| Gang Li, PhD, MD | Shanghai East Hospital, Tongji University School of Medicine, Shanghai, China | Site Investigator | Data collection |
